# Evaluation of Partial Breast Reirradiation with Intraoperative Radiotherapy after Prior Thoracic Radiation: A Single-Institution Report of Outcomes and Toxicity

**DOI:** 10.3389/fonc.2017.00175

**Published:** 2017-08-28

**Authors:** Christine Chin, Priya Jadeja, Bret Taback, David P. Horowitz, Sheldon M. Feldman, Richard Ha, Eileen P. Connolly

**Affiliations:** ^1^Radiation Oncology, NYP-Columbia University Medical Center, New York, NY, United States; ^2^Breast Surgery, NYP-Columbia University Medical Center, Herbert Irving Pavilion, New York, NY, United States; ^3^Breast Surgery and Breast Surgical Oncology, Montefiore M-E Center for Cancer Care, Bronx, NY, United States; ^4^Radiology, NYP-Columbia University Medical Center, Herbert Irving Pavilion, New York, NY, United States

**Keywords:** breast, accelerated partial breast irradiation, intraoperative radiation therapy, recurrence, reirradiation, toxicity

## Abstract

**Introduction:**

Mastectomy is the current standard of care for ipsilateral breast tumor recurrences after prior whole breast irradiation (WBI). We report our single-institution experience with breast-conserving surgery (BCS) followed by intraoperative radiotherapy (IORT) as an alternative to salvage mastectomy for new or recurrent breast cancers that develop in the setting of prior thoracic radiation.

**Methods:**

We performed an IRB-approved retrospective review of patients treated with breast IORT between September 2013 and November 2016. We identified 12 patients who declined salvage mastectomy for their breast cancer after prior thoracic radiation. IORT was delivered using the Intrabeam™ device (Carl Zeiss, Germany). A dose of 20 Gy was prescribed to the lumpectomy cavity surface using 50 kV X-rays. We graded both acute and late treatment-related breast toxicities using the National Cancer Institute Common Terminology Criteria for Adverse Events version 4.0. Local control, mastectomy-free survival, distant metastasis, and overall survival were determined.

**Results:**

Our study included nine patients who developed a new or recurrent ipsilateral breast cancer after prior WBI for early-stage breast cancer, two patients with primary breast cancer after mantle-field radiation for Hodgkin’s lymphoma, and one patient with a synchronous stage III non-small cell lung cancer treated with definitive radiation to the ipsilateral lung and mediastinum. The median time from prior radiation to presentation was 18 years (range: 2 months to 46 years). All patients successfully underwent partial breast reirradiation with IORT and were able to preserve their breast. At a median follow-up of 14 months (4–25 months), there were no local or distant recurrences. There was a single non-cancer-related death. In the acute setting, we observed grade 1 toxicity in 58% (*n* = 7), grade 2 toxicity in 17% (*n* = 2), and no grade 3 or higher toxicity. In the late setting, at least 3 months after IORT, we observed grade 1 hyperpigmentation and/or fibrosis in 50% (*n* = 6), symptomatic seroma requiring drainage in 33% (*n* = 4). A single patient developed an abscess requiring hospitalization and intravenous antibiotic therapy.

**Conclusion:**

BCS with IORT is a feasible salvage option for patients who present with localized breast cancer after prior thoracic radiation treatment. Continued follow-up of these patients is warranted given the incidence of delayed toxicity.

## Introduction

Based on a number of large randomized trials, the estimated 10-year rate of an isolated ipsilateral breast tumor recurrence (IBTR) after breast-conserving therapy is approximately 10% (1, 2). At the time of local recurrence the current standard of care is salvage mastectomy given the unacceptable toxicity to normal tissues with repeat whole breast irradiation (WBI). Local excision alone of an IBTR results in subsequent local recurrence in approximately 35% of patients based on retrospective series (3–8). Interestingly, in women who develop late local recurrences more than 5 years after treatment of their primary disease, disease-free, and overall survival is not significantly different compared to women who do not experience an IBTR (4, 9, 10). The prolonged interval until recurrence reflects a favorable tumor biology, and retrospective studies report no difference in survival between patients who undergo salvage mastectomy and breast-conserving surgery (BCS) for small, localized recurrences (10, 11). Given that many IBTRs are detected early on surveillance-imaging, many patients desire a breast-conserving option at the time of recurrence. Breast-conserving therapies have therefore become increasingly popular in treating these patients.

Accelerated partial breast irradiation (APBI) is a novel technique that offers the opportunity to limit radiation dose to previously irradiated breast tissue while improving rates of local control after BCS (12–16). There have been a limited number of small retrospective and prospective studies examining the use of APBI after local excision of an IBTR in the setting of prior radiation using various dose fractionations and delivery techniques (17–23). The use of intraoperative radiation therapy (IORT) to deliver a single, high-dose radiation to the lumpectomy surface at the time of surgery has been compared to adjuvant whole breast radiotherapy in the treatment of unifocal, early-stage breast cancers with non-inferior results (13). Its use in the setting of reirradiation has been reported with acceptable toxicity and cosmesis in small retrospective studies (13, 24, 25). Here, we report our single-institution experience with partial breast reirradiation (PBrI) with IORT after BCS in patients who decline salvage mastectomy.

## Materials and Methods

### Patient Eligibility

We performed a retrospective review approved by the Columbia University Institutional Review Board of 228 patients treated with breast IORT between September 1, 2013, and November 31, 2016. Written informed consent was obtained from research participants. Patients were included in this study if they had developed a unifocal IBTR or new primary breast cancer (PBC) in the setting of prior WBI for early-stage breast cancer or a PBC after definitive thoracic radiation for another primary malignancy and declined salvage mastectomy. IBTR was defined as a breast tumor recurrence with the same histology and location as the initial PBC.

### Radiation Treatment

Intraoperative radiotherapy was delivered using the Intrabeam™ device (Carl Zeiss, Oberkochen, Germany) at the time of lumpectomy. A spherical applicator was chosen at the radiation oncologist and operating surgeon’s discretion to most appropriately fit the lumpectomy cavity. Ultrasound was used to confirm a minimum skin to applicator margin of at least 10 mm. A medical physicist was present to confirm delivery of 20 Gy to the surface of the lumpectomy cavity using 50 kV X-rays.

### End Point Analysis

Patients were encouraged to follow-up at 2 weeks after surgery and at least every 6 months for the first year after treatment. End points of local control, mastectomy-free survival, distant metastasis, and overall survival were determined from the time of IORT. Acute and long-term side effects including breast pain, dermatitis, fibrosis, seroma, and infection were graded according to the National Cancer Institute Common Terminology Criteria for Adverse Events (CTCAE) version 4.0.

## Results

### Patient and Primary Tumor Characteristics

The median age of our patients at the time of recurrence was 65 years old (range: 52–85 years). The median time from prior external beam radiation to biopsy-proven recurrence was 18 years (range: 2 months to 46 years). Nine patients developed an IBTR or new PBC after prior WBI for early-stage breast cancer, 2 patients with a new PBC after mantle-field radiation for Hodgkin’s lymphoma, and 1 patient with a synchronous diagnosis of stage III non-small cell lung cancer (NSCLC) treated with definitive radiation to the ipsilateral lung and mediastinum. Only one patient with the synchronous diagnosis of stage III NSCLC and breast cancer underwent breast IORT at an interval of less than 1 year from the time of prior definitive radiation. Further available details of patient primary tumor and treatment characteristics are presented in Table 1.

**Table 1:** Primary tumor and treatment characteristics.

Patient	Age at initial diagnosis (years)	Histology of primary disease	Subtype	Prior radiation details	Prior adjuvant therapy
1	53	IDC	ER/PR+	WBI 50 and 15 Gy boost	CMF, tamoxifen
2	38	Hodgkin’s lymphoma	–	Mantle field 40 Gy	–
3	44	DCIS	ER/PR+	WBI dose unknown	Tamoxifen
4	76	IDC	ER/PR+	WBI dose unknown	Anastrazole started but did not tolerate
5	49	DCIS	ER/PR+	WBI 50 and 15 Gy boost	None
6	52	NSCLC	–	Right lung and mediastinum IMRT 66 Gy^b^	–
7	43	IDC	TNBC	WBI 50.4 and 10 Gy boost	ddAC
8	20	Hodgkin’s lymphoma	–	Mantle field 40 Gy	–
9	53	DCIS	ER/PR−	WBI 50.4 and 10 Gy boost	None
10	51	IDC	ER/PR+	WBI, 50.4 and 12 Gy boost^a^	A/T/bevacizumab/lupron^b^
11	59	IDC	ER/PR+	WBI dose unknown	Tamoxifen
12	54	IDC	ER/PR+	WBI dose unknown	None

*IDC, invasive ductal carcinoma; ER, estrogen receptor; PR, progesterone receptor; NSCLC, non-small cell lung cancer; IMRT, intensity modulated radiation therapy; CMF, cyclophosphamide, methotrexate, fluorouracil; ddAC, dose dense doxorubicin, cyclophosphamide; AT, doxorubicin, paclitaxel*.

*^a^Patient was treated with neoadjuvant chemotherapy and definitive radiation therapy for her initial primary. ^b^Therapy given in the neoadjuvant setting*.

*^b^Right breast mean dose 10.9 Gy, max point dose 59.6 Gy*.

### Pathologic Findings

Based on final pathology, the median tumor size was 0.55 cm (range: 0–3.9 cm). All patients with invasive primaries were hormone receptor positive, defined as ≥1% staining of the estrogen and/or progesterone receptor by immunohistochemistry (26). Two patients were HER2/neu positive per American Society of Clinical Oncology/College of American Pathologists (ASCO/CAP) guideline recommendations (27). One of two patients with ductal carcinoma in situ (DCIS) was hormone receptor negative. Five patients underwent axillary sampling at the time of surgery for their IBTR. One patient was found to have involved sentinel lymph nodes and underwent completion of an axillary lymph node dissection. All patients with invasive primaries were found to have negative margins with no tumor on the final inked margin. One patient with DCIS was found to have a final close margin of 1 mm with no further re-excision. A summary of these findings is shown in Table 2.

**Table 2:** Patient treatment characteristics.

Patient	Age at recurrence (years)	Time to recurrence^b^ (years)	Histology of breast tumor	Type of recurrence	Subtype	IBTR size (cm)	Lymph node sampling	Adjuvant systemic therapy
1	78	25	IDC	IBTR	ER/PR+	0.2	0/3	Exemestane
2	74	36	IDC	PBC	PR+	0.2	–	Intolerance of AI
3	60	16	DCIS	IBTR	PR+	0^a^	–	None
4	85	9	IDC	IBTR	ER/PR+	0.4	–	None, previous intolerance of AI
5	78	29	DCIS	IBTR	ER/PR+	0.6	–	Tamoxifen
6	52	0.2	IDC	PBC	ER/PR+	3.5	5/20	TC, anastrazole
7	64	21	IDC	SBC	PR+	1.1	0/2	Anastrazole
8	66	46	DCIS	PBC	ER/PR−	0.5	–	None
9	64	11	IDC	SBC	ER/PR+	1.5	0/3	Anastrazole but did not tolerate
10	55	5	Mixed IDC/ILC	IBTR	ER/PR+	1.9	0/14	Letrozole
11	78	19	IDC	SBC	ER/PR+, HER2+	3.9	–	Anastrazole, trastuzumab
12	62	8	IDC	SBC	ER/PR+, HER2+	0.4	0/1	Anastrazole, trastuzumab

*IBTR, ipsilateral breast tumor recurrence; SBC, second breast cancer defined as an ipsilateral breast cancer of a different histology from the initial primary breast cancer; PBC, primary breast cancer in the setting of another prior malignancy; AI, aromatase inhibitor; TC, docetaxel and cyclophosphamide*.

*^a^Patient 3 had no residual disease at the time of lumpectomy*.

*^b^Interval time from prior diagnosis to biopsy-proven breast recurrence*.

### IORT Details

The median applicator size used in our cohort of patients was 3.0 cm (range: 3.0–3.5 cm). All patients were prescribed 20 Gy to the lumpectomy surface using 50 kV X-rays. The median treatment time was 24.2 min (range: 17.1–24.7 min). The median applicator to skin distance was superiorly 15.3 mm (range: 5.1–23.0 mm), inferiorly 13.9 mm (range: 8.3–21.9 mm), medially 19.4 mm (range: 11.0–29.7 mm), and laterally 13.9 mm (range: 6.3–22.1 mm).

### Adjuvant Systemic Therapy

All patients with hormone receptor-positive disease were recommended adjuvant hormonal therapy. All patients were compliant with therapy except three patients due to intolerance. One patient with disease metastasis to the axilla was treated with adjuvant docetaxel and cyclophosphamide chemotherapy. Two patients with HER2-positive invasive disease received adjuvant trastuzumab therapy.

### Outcomes and Toxicity

At a median follow-up of 14 months (range: 4–25 months) there were no events of local or distant recurrence, and all women were able to preserve their breast. There was a single non-breast cancer-related death due to heart failure. In the acute setting, we observed grade 1 dermatitis in 25% (*n* = 3), grade 1 breast pain in 8% (*n* = 1), grade 1 seroma in 25% (*n* = 3), grade 2 seromas requiring drainage in 17% (*n* = 2), and grade 2 infection in 8% (*n* = 1). No grade 3 or higher acute toxicity was observed. In the late setting, defined as 3 months after treatment with IORT, we observed grade 1 hyperpigmentation and/or fibrosis in 50% (*n* = 6), grade 1 seromas in 8% (*n* = 1), persistent grade 2 seromas in 33% (*n* = 4), and grade 2 infection in 17% (*n* = 2). There was a single patient who developed a grade 3 abscess requiring hospitalization and intravenous antibiotic therapy. There were no grade 2 or higher toxicity for the patient who underwent breast IORT within a year of prior definitive RT. Details of patient toxicity regarding breast seroma formation and infection are presented in Table 3.

**Table 3:** CTCAE graded treatment toxicity.

Patient	Dermatitis (acute)	Skin changes (late)	Breast infection (acute)	Breast infection (late)	Seroma (acute)	Seroma (late)	Fibrosis (acute)	Fibrosis (late)	Breast pain (acute)	Breast pain (late)
1	0	0	0	0	0	1	0	0	0	0
2	0	0	0	0	0	0	0	0	0	0
3	0	0	0	0	0	0	0	1	0	0
4	0	0	0	2	1	2	0	0	0	0
5	1	0	2	0	2	0	0	1	0	0
6	0	0	0	0	0	0	0	0	0	0
7	1	0	2	2	2	2	0	0	1	0
8	1	0	0	0	0	0	0	0	0	0
9	0	1	0	0	1	2	0	1	0	0
10	0	1	0	3	1	2	0	1	0	0
11	0	1	0	0	0	0	0	0	0	0
12	0	0	0	0	0	0	0	1	0	1

Total	25%	25%	17%	25%	42%	42%	0	42%	8%	8%

*All toxicity graded per the NCI CTCAE version 4.02; acute defined as within 3 months from intraoperative radiotherapy treatment*.

## Discussion

In our single-institution experience, all 12 of our patients were able to successfully undergo local excision of their breast cancer followed by IORT with acceptable toxicity and preserve their breast. After a median follow-up of 14 months, there were no events of local or distant recurrence. In the late setting after treatment, we observed a significant incidence of persistent grade 2 seromas requiring drainage and antibiotic therapy. In the current literature, there are conflicting accounts of post-APBI seroma formation using various brachytherapy techniques. Evans et al. reported persistent seromas in about 75% of patients (greater than 6 months after treatment) treated with APBI using MammoSite^®^. Evaluating various dosimetric, clinical, and treatment-related variables, higher body weight was the only significant variable that correlated positively with seroma formation (28). On the contrary, Kraus-Tiefenbacher reported no difference in the rate of palpable seromas between patients who underwent BCS with or without IORT at the time of lumpectomy. While radiographically detected seromas were higher in the IORT group (81 vs. 52%, *p* < 0.001), the rate of palpable clinically significant seromas was not different. In their analysis, the addition of adjuvant chemotherapy correlated with higher rates of seromas detected on follow-up CT imaging (contingency coefficient 0.22, *p* = 0.003) (29). The higher incidence of seromas we observed in our study may reflect the lower tolerance of normal tissue in the reirradiation setting.

There has been one other published report of PBrI with IORT in a cohort of 17 patients who developed localized breast recurrences after previous external beam radiation (25). Overall, with a median follow-up of 26 months there were no reported local recurrences. Acute toxicity consisted mainly of mild induration of the tumor bed, and there were no instances of grade 3 or 4 toxicity. Interestingly, in comparison to our study, a larger median size applicator was used to treat the lumpectomy cavity (median 4.0 cm, range: 2.5–5.0 cm) and 3 of the 17 patients treated with ≥4.5 cm applicator were prescribed a lower dose of 14.7 Gy to the lumpectomy surface. While both of our cohorts are small, the difference in our reported experiences highlights the importance of applicator selection. Larger applicator selection may help to optimize apposition of the applicator against surrounding breast tissue and improve dose homogeneity at the tissue–applicator interface.

There are a number of single-institution studies that have reported on the toxicity and outcomes of PBrI. Deutsch et al. reported on their experience with PBrI using external beam electrons to cover the involved breast quadrant to 50 Gy in 2 Gy per daily fraction. They reported a recurrence free rate of 77% at 52-month follow-up, and overall good cosmesis with mainly skin pigmentation changes (14). Interstitial brachytherapy has been used by several institutions, reporting late toxicity of grade 3 fibrosis in up to 10–16% of patients (15, 17, 19, 20). Freedom from a second local recurrence at a median follow-up of 5 years was 89–93%. Existing studies support the efficacy and safety of PBrI to treat IBTR. To date, however, there is no clear optimal delivery technique or dose fractionation. RTOG 1014 is the most recent prospective trial examining the safety and efficacy of PBrI for IBTR after prior WBI using 3D-conformal external beam radiation. Their preliminary outcomes were reported at the recent American Society for Radiation Oncology conference, describing a 3-year subsequent IBTR of 3.7%, DMFS and OS of 94.8% in a cohort of 58 patients. Four patients underwent subsequent mastectomy, two for a subsequent IBTR, one for a non-healing wound and another patient who underwent bilateral mastectomy after discovery of contralateral disease (30). They describe grade 1 late toxicity (greater than 1 year from treatment) in 24.1% of patients mainly consisting of breast pain and fibrosis, grade 2 late toxicity in 22.4%, and grade 3 toxicity in 6.9% with one instance of grade 3 infection. Comparable to the 3-year toxicity data from RTOG 1014, we observed a significant number of grade 2 and 3 toxicities in the late setting.

### Limitations

The major limitations of this study include the retrospective nature of our data with limited follow-up to observe further delayed toxicities and recurrences. Given the small sample population of our study, it is difficult to draw conclusions regarding the differences in toxicity between our PBrI experience and previously published studies. Finally, we did not report on patient satisfaction and quality of life in our study. The pursuit of breast-conservation was driven mainly by patient preference, and ultimately the low-grade toxicities we observed with breast conservation may be outweighed by the success of breast preservation.

## Conclusion

Breast-conserving surgery with IORT is a feasible salvage option for patients desiring breast conservation after prior thoracic radiation; however, continued follow-up of these patients is warranted given the incidence of delayed treatment toxicity. Further studies are needed to determine optimal treatment strategy, dose, and dose fractionation for PBrI.

## Author Contributions

Each of the listed authors contributed to the completion of this submission. Both CC and PJ worked on data collection and drafting of the final manuscript. BT, SF, RH, DH, and EC all contributed to the critical revision of the article with final approval for publication.

## Conflict of Interest Statement

The authors declare that the research was conducted in the absence of any commercial or financial relationships that could be construed as a potential conflict of interest.
